# 75561 Association of childhood hypertension with early adulthood obesity and hypertension

**DOI:** 10.1017/cts.2021.486

**Published:** 2021-03-30

**Authors:** Mindy Pike, T. Alp Ikizler, Loren Lipworth, Cassianne Robinson-Cohen

**Affiliations:** Vanderbilt University Medical Center

## Abstract

ABSTRACT IMPACT: This study establishes the association between childhood hypertension and health outcomes in early adulthood, identifying the need to understand blood pressure during early life for primary prevention of hypertension and cardiovascular disease. OBJECTIVES/GOALS: There is evidence that blood pressure level in early life can influence hypertension and other cardiovascular risk factors later in life. We examined whether hypertension before the age of 18 is associated with higher odds of obesity and hypertension after the age of 18. METHODS/STUDY POPULATION: We studied 19,367 children and adolescents from the Vanderbilt University Medical Center’s Synthetic Derivative, a de-identified version of the electronic medical record. Childhood hypertension was defined as systolic blood pressure (SBP) ≥130 mmHg or diastolic blood pressure (DBP) ≥80 mmHg at three or more outpatient visits before the age of 18. Obesity and hypertension in early adulthood were the primary outcomes. Obesity was defined as being above normal weight for adulthood height at age 30 based on the NIH’s body mass index tables. Hypertension was defined as SBP ≥130 mmHg or DBP ≥80 mmHg at three or more outpatient visits after the age of 18. Odds ratios and 95% confidence intervals (CIs) were computed from logistic regression models adjusted for demographics, medication use, and childhood weight. RESULTS/ANTICIPATED RESULTS: Most subjects were female (63%) and white (80%). During childhood, 17% of participants had hypertension. Approximately 58% of this group were obese at age 30, and 38% had hypertension as adults. Compared to females with no childhood hypertension, females with childhood hypertension had 1.35 times higher odds of being obese at age 30 (95% CI: 1.15, 1.58) and 3.56 times higher odds of having hypertension over the age of 18 (95% CI: 3.09, 4.09). Males with childhood hypertension, compared to males without, had 1.28 times higher odds of being obese at age 30 (95% CI: 1.08, 1.52) and 2.74 times higher odds of having hypertension over the age of 18 (95% CI: 2.35, 3.20). Associations between childhood hypertension, early adulthood obesity, and hypertension significantly differed by gender (p-for-interaction for both: <0.01). DISCUSSION/SIGNIFICANCE OF FINDINGS: Childhood hypertension is associated with obesity and hypertension in early adulthood. Understanding blood pressure levels in childhood and adolescence may help target efforts to reduce early adulthood cardiovascular risk factors.

